# ABCG2 Transports the Flukicide Nitroxynil and Affects Its Biodistribution and Secretion into Milk

**DOI:** 10.3390/pharmaceutics16040558

**Published:** 2024-04-19

**Authors:** Laura Álvarez-Fernández, Esther Blanco-Paniagua, Gracia Merino

**Affiliations:** Department of Biomedical Sciences-Physiology, Faculty of Veterinary Medicine, Animal Health Institute (INDEGSAL), University of León, Campus de Vegazana s/n, 24071 León, Spain; lalvf@unileon.es (L.Á.-F.); eblap@unileon.es (E.B.-P.)

**Keywords:** ABC transporter, biological barriers, milk secretion, nitroxynil, pharmacokinetics, tissue distribution

## Abstract

The ABCG2 transporter plays a key role in pharmacological and toxicological processes, affecting bioavailability, tissue accumulation and milk secretion of its substrates. This protein is expressed in several biological barriers acting as a protective mechanism against xenobiotic exposure by pumping out a broad range of compounds. However, its induced expression during lactation in alveolar cells of mammary gland represents a relevant route for active transport of unwanted chemicals into milk. This work aimed to characterize the involvement of ABCG2 in systemic exposure and milk secretion of the flukicide nitroxynil. Using MDCK–II cells overexpressing the transporter, we showed that nitroxynil is an in vitro substrate of different species variants of ABCG2. Moreover, using wild-type and Abcg2^−/−^ mice, we showed that murine Abcg2 clearly affects plasma levels of nitroxynil. We also reported differences in nitroxynil accumulation in several tissues, with almost 2-fold higher concentration in kidney, small intestine and testis of Abcg2^−/−^ mice. Finally, we proved that nitroxynil secretion into milk was also affected by Abcg2, with a 1.9-fold higher milk concentration in wild-type compared with Abcg2^−/−^ mice. We conclude that ABCG2 significantly impacts nitroxynil biodistribution by regulating its passage across biological barriers.

## 1. Introduction

ABCG2 is one of the most important ABC efflux proteins involved in pharmacological and toxicological processes by pumping a wide range of compounds out of the cells. It is expressed in the apical membrane of polarized cells from several tissues such as the luminal membrane of enterocytes, the bile canalicular membrane of hepatocytes and the renal proximal tubule epithelia, among others [1]. These locations have been associated with a protective role for ABCG2 since it restricts the absorption of its substrates in the intestine, limits their oral availability and regulates their tissue distribution and hepatobiliary and renal excretion, thus affecting systemic exposure and pharmacokinetics of numerous drugs and natural compounds [2]. This membrane transporter is also present in other biological barriers, such as blood–brain, blood–testis and blood–fetal barriers [3,4,5], limiting the brain, testicular and fetal penetration and accumulation of its substrates. Paradoxically, ABCG2 expression is also induced during lactation in the apical membrane of alveolar epithelial cells of mammary gland, acting as a relevant route for active transport of unwanted chemicals into milk. As a result, it increases the risk for milk contamination with xenobiotic residues [6] and the potential exposure of sucking neonates and consumers of dairy products to harmful substances [7]. An evolutionary rationale for this paradox between protective function of ABCG2 and its role in milk contamination has been widely discussed previously [8].

Among the drugs that interact with ABCG2 are some parasiticides [9]. Nevertheless, the interaction between the parasiticide nitroxynil and ABCG2 remains unexplored. Nitroxynil is a veterinary drug commonly used for prophylaxis and treatment of fascioliasis, a zoonotic disease that significantly affects growth, reproduction, and milk and meat production in food-producing cattle and sheep. It is also effective against some gastrointestinal roundworms [10,11]. 

Nowadays, fasciolicides are the key strategy for controlling fascioliasis in livestock and human medicine. However, anthelmintic resistance is a global problem of increasing concern and the overuse of a particularly effective treatment such as triclabendazole is inevitably leading to resistance problems and, thus, nitroxynil may be a relevant alternative [12], both administered alone and in combination with other fasciolicides [13,14]. Although compared with other flukicides, nitroxynil demonstrates higher efficacy against both mature and immature stages of flukes [15], its long body persistence requires special attention. In fact, nitroxynil residues have been found after treatment up to 58 days in milk [16] and 90 days in tissues [17], subsequently endangering human health and food safety through the food chain due to potential unexpected residues in foodstuffs. Therefore, the European Commission Regulation has established the maximum residue limit (MRL) for nitroxynil in different tissues (20–400 µg/kg) and, more recently, in ruminant milk (20 µg/kg) [18] with long withdrawal periods. However, despite this strict regulation, the presence of this drug has been reported in human breast milk from mothers untreated with nitroxynil [19]. 

The purpose of this work was to assess the potential role of ABCG2 in plasma levels, tissue accumulation and active secretion into milk of nitroxynil using wild-type and Abcg2^−/−^ mice. In vitro transport studies with cells overexpressing the transporter were used to correlate in vitro and in vivo results.

## 2. Materials and Methods

### 2.1. Chemicals

Reference standard for nitroxynil was purchased from LGC Standards (Teddington, Middlesex, UK). Nitrofurantoin and Lucifer Yellow were supplied by Sigma–Aldrich (St. Louis, MO, USA). Ko143 was obtained from Tocris (Bristol, UK). For in vivo studies, nitroxynil (Distomicide^®^ 250 mg/mL) was acquired from Laboratorios Ovejero S.A. (León, Spain), oxytocin (Facilpart^®^) from Syva (León, Spain) and isoflurane (Isovet^®^) from Braun VetCare (Barcelona, Spain). All the other chemicals used were of analytical grade and available from commercial sources.

### 2.2. Cell Cultures

Parental Madin–Darby canine kidney epithelial cells (MDCK–II) and their subclones transduced with murine (mAbcg2) and human (hABCG2) variants of ABCG2 were kindly provided by Dr. A. H. Schinkel (Netherlands Cancer Institute). Transduced MDCK–II cells with the ovine (oABCG2) and bovine (bABCG2) variants were previously generated by our group. Cells were cultured as described elsewhere [9].

#### Transport Studies

Transepithelial transport assays were carried out as previously described [9] with minor variations, using Hanks’ balanced salt solution (Sigma-Aldrich) supplemented with HEPES (25 mM), with or without Ko143 (1 μM), and containing or not nitroxynil 10 μM. At 1, 2 and 3 h, 100 μL aliquots were collected from the opposite compartment where nitroxynil was added, and at 4 h, 600 µL aliquots were taken from both sides of the monolayer. Aliquots were stored at −20 °C until processing. At the end of the experiment, confluence of the monolayer was measured with the previously described Lucifer Yellow permeability test [20], with minor modifications. The appearance of nitroxynil in the opposite side of the well was measured by HPLC as later described. Active transport was expressed as relative efflux transport ratio (apically directed transport percentage divided by the basolaterally directed translocation percentage) at 4 h and referred to the initial concentration. Transport proficiency of cells was simultaneously checked by analyzing a typical ABCG2 substrate (danofloxacin 10 μM) [21].

### 2.3. Animals

All in vivo experiments were carried out using wild-type and Abcg2^−/−^ mice aged between 8 and 17 weeks, all of >99% FVB/N genetic background, generated and gently supplied by A. H. Schinkel (Netherlands Cancer Institute). Mice were housed and handled complying with institutional and ARRIVE guidelines and European legislation (EU Directive 2010/63/EU for animal experiments) [22]. Additionally, experimental procedures were approved by the Animal Care and Use Committee of the University of León and the Junta de Castilla y León (ULE_011_2019).

#### 2.3.1. Pharmacokinetic Experiments

For pharmacokinetic experiments, a single dose of nitroxynil at 10 mg/kg was administered subcutaneously to wild-type and Abcg2^−/−^ male mice (*n* = 4–5, for each time point). Distomicide^®^ (250 mg/mL) commercial solution was dissolved in saline (sodium chloride 0.9%) and dosed at 200 μL per 30 g body weight. Blood samples were collected at different time points (0.5, 3, 4.5, 6, 24, 48 and 96 h) and heparinized blood samples were centrifugated for 15 min at 3000× *g*. Organs were also collected at 24 and 96 h after euthanasia. Linear trapezoidal rule was used to calculate the area under the plasma concentration–time curve (AUC) parameter.

#### 2.3.2. Milk Secretion Experiments

Pups of approximately 10 days old were separated from their mothers (wild-type (*n* = 10) and Abcg2^−/−^ (*n* = 11) female mice) 3 h before beginning the experiment. Milk secretion experiments were performed as previously described [9]. Nitroxynil solution was prepared by diluting Distomicide^®^ (250 mg/mL) commercial solution in saline and administered at a dose of 150 μL/30 g bw into the tail vein under anesthesia with isoflurane. An amount of 200 μL oxytocin (1 IU/mL) was administrated subcutaneously 10 min before milk collection. Blood and milk samples were collected from retro-orbital sinus and mammary gland, respectively, 30 min after nitroxynil administration.

### 2.4. Sample Preparation for HPLC Analysis

Plasma, milk and tissue samples from each animal were individually processed and analyzed without pooling, while samples from transport assays were not processed. Tissue samples were homogenized in a mixture of 50% water/50% methanol HCl 0.5% (*v*/*v*); 1 mL solution per 0.1 g tissue. The entire amount of collected milk (74.7–295.1 mg) and 100 μL plasma and tissue homogenates were processed. To each sample, 10 μL of a nitrofurantoin solution (10 μg/mL for plasma and milk and 2 μg/mL for homogenized tissues) was added as an internal standard and 150 μL acetonitrile was used to precipitate the proteins. The mixture was vortexed horizontally for 15 min and centrifugated at 10,000× *g* for 5 min at 4 °C. Supernatant was transferred to a new tube and dried at 40 °C under N_2_ stream. Samples were reconstituted in 100 μL of mobile phase and 50 μL was injected into HPLC. Standards were subjected to the same procedures as samples.

### 2.5. Chromatographic Analysis by HPLC 

Waters 2695 separation module and Waters 2998 UV photodiode array detector set at 270 nm were used for samples analysis. Chromatographic conditions for nitroxynil detection were based on previously described methods [23], with minor modifications. Chromatographic separation was achieved on a reverse-phase column (Synergy 4 μm Hydro–RP 80 Å, 250 × 4.60 mm; Phenomenex^®^, Torrance, CA, USA) using an isocratic binary mobile phase consisting of 25 mM potassium phosphate monobasic (KH_2_PO_4_), pH 4: acetonitrile (70:30 for culture samples and 80:20 for animal samples) at a flow rate of 1 mL/min. Autosampler and column temperature were 4 °C and 40 °C, respectively. Limit of detection (LOD) and quantification (LOQ) were calculated as previously described [24]. For cell culture samples, LOD and LOQ were 0.003 μg/mL and 0.007 μg/mL, respectively. For in vivo samples, LOD was in the range of 0.01–0.07 μg/mL and LOQ in the range of 0.03–0.15 μg/mL.

### 2.6. Statistical Analysis

Comparisons between groups were performed using the two-tailed unpaired Student’s *t*-test (normally distributed variables) and the non-parametric Mann–Whitney U test (not-normally distributed variables). The normal distribution of the variables was checked applying the Shapiro–Wilk normality test. All analysis were carried out on the assumed significance level of *p* ≤ 0.05 using SPSS Statistics software v. 26 (IBM, Armonk, NY, USA). The results are shown as mean ± standard deviation (SD).

## 3. Results

### 3.1. In Vitro Transport of Nitroxynil

**Figure 1:**
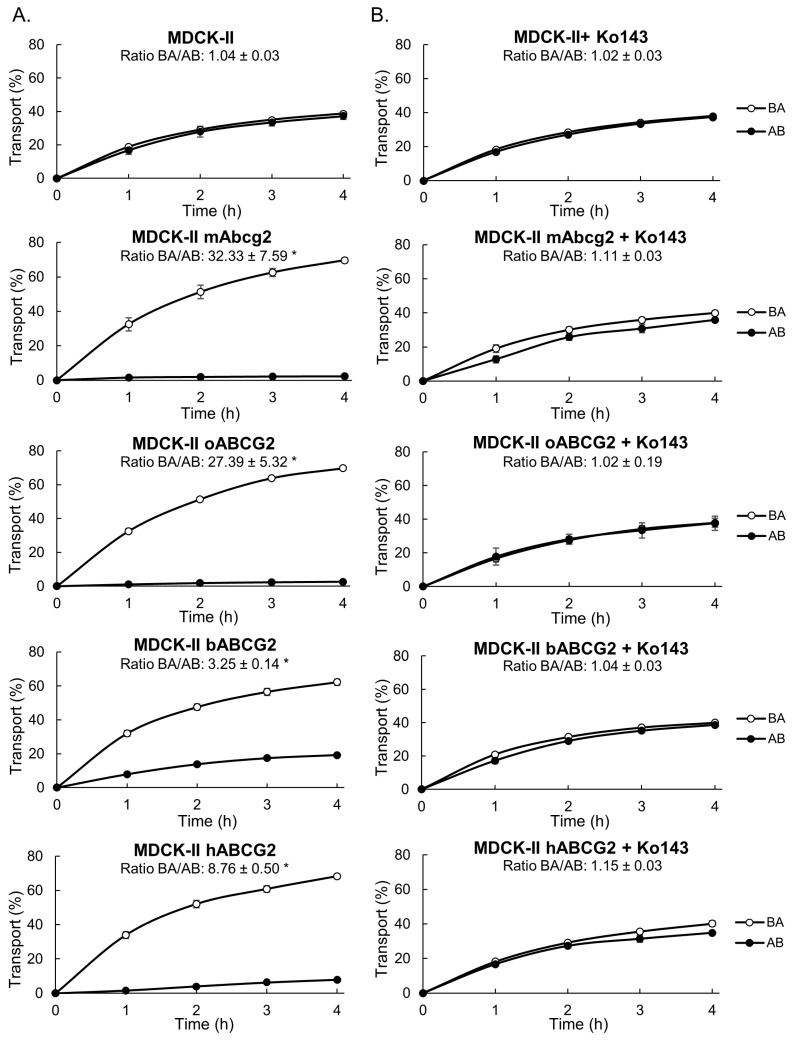
Transepithelial in vitro transport of nitroxynil at 10 µM in the absence (**A**) or presence (**B**) of the specific inhibitor of ABCG2, Ko143 (1 µM) in MDCK-II cells overexpressing murine, ovine, bovine and human variants of the ABCG2 transporter (mAbcg2, oABCG2, bABCG2 and hABCG2, respectively). Nitroxynil concentration at different time points was determined by HPLC and transport across monolayers was related to the total drug added at beginning of the experiment in the donor compartment_._ Results are presented as means and error bars indicate SD. The relative efflux transport ratio (apically directed transport percentage divided by the basolaterally directed translocation percentage) at 4 h is indicated. (*) *p* ≤ 0.05, significant differences in transport ratio compared to parental MDCK–II cells (Student’s *t*-test, normally distributed data) (*n* = 3–8).

The ABCG2 role in the in vitro transport of nitroxynil was determined using MDCK–II polarized cells. Parental cell line (without ABCG2 transduction) and its transduced subclones were grown to form confluent monolayers that were used to evaluate vectorial transport of nitroxynil. A similar transport pattern with equal apical (apical to basal directed transport, AB) and basolateral (basal to apical directed transport, BA) vectorial translocation was observed in the parental MCDK–II cell line (Figure 1A). In the case of murine Abcg2 and ovine ABCG2-transduced cells and, to a lesser extent, in the bovine and human variants, BA directed transport of nitroxynil highly increased and AB translocation notably decreased compared with the MDCK–II parental cell line (Figure 1A). Relative efflux transport ratio (BA/AB, at 4 h) was significantly higher in transduced subclones related to parental cells (32.33 ± 7.59 for murine Abcg2, 27.39 ± 5.32 for ovine ABCG2, 3.25 ± 0.14 for bovine ABCG2 and 8.76 ± 0.50 for human ABCG2 vs. 1.04 ± 0.03 for parental MDCK–II cells; *p* < 0.05). In all cases, the specificity of ABCG2-mediated transport was checked using the ABCG2 inhibitor Ko143. The ABCG2-mediated transport was inhibited for all types of transduced cells (Figure 1B) and relative efflux ratios equal to parental cells were obtained in the ABCG2-transduced cells. These results demonstrate that nitroxynil is an efficient in vitro substrate of ABCG2.

### 3.2. Plasma Pharmacokinetics of Nitroxynil in Wild-Type and Abcg2^−/−^ Male Mice

**Figure 2:**
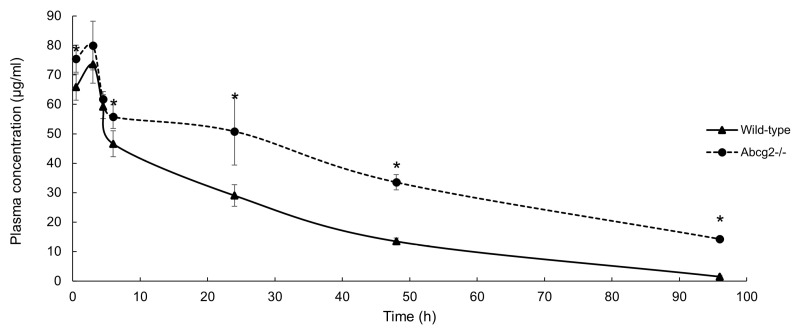
Plasma concentration of nitroxynil in wild-type and Abcg2^−/−^ male mice at 0.5, 3, 4.5, 6, 24, 48 and 96 h after subcutaneous administration of Distomicide^®^ at a dose of 10 mg/kg body weight. Results are presented as means and error bars indicate SD. (*) *p* ≤ 0.05, significant differences compared to wild-type mice (Student’s *t*-test, normally distributed data) (*n* = 4–5).

Plasma pharmacokinetics was performed with the aim of corroborating whether in vitro Abcg2-mediated transport of nitroxynil was reflected in the vivo situation. Nitroxynil concentration was determined in both type of mice at a dose 10 mg/kg, and expressed as a function of time (Figure 2).

The drug was rapidly absorbed with the C_max_ at the second sampling point (3 h) for both type of mice and no significant differences were reported. Subsequently, the concentration decreased rapidly during the following 3 h and then decreased as a slower rate. Notably, significant differences in plasma concentration of nitroxynil between both type of mice were found at several time points (0.5 h, *p* = 0.02; 6 h, *p* = 0.02; 24 h, *p* = 0.01; 48 h, *p* < 0.001 and 96 h, *p* < 0.001) (Figure 2). The differences observed in plasma concentration versus time profiles between groups were confirmed by the AUC parameter. Plasma AUC of Abcg2^−/−^ was 1.8-fold higher compared with wild-type mice (3528.0 ± 129.5 μg·h/mL vs. 1922.1 ± 49.1 μg·h/mL, respectively, *p* = 0.01). These results further substantiate that nitroxynil is an in vivo substrate of Abcg2 and clearly show that Abcg2 affects plasma pharmacokinetics of nitroxynil. 

### 3.3. Effect of Abcg2 in Tissue Distribution of Nitroxynil

Nitroxynil concentration was analyzed in several tissues, including liver, kidney, small intestine, spleen, testis and brain, from wild-type and Abcg2^−/−^ male mice, at 24 and 96 h after subcutaneous administration of 10 mg/kg of the drug (Table 1). Regardless of Abcg2 expression, tissue levels of nitroxynil were higher at 24 h than at 96 h, with the highest amounts in the kidney and testis at both sampling time points. In the absence of Abcg2, tissue accumulation of nitroxynil was higher than in wild-type mice. Specifically, Abcg2^−/−^ mice showed 1.4-fold higher levels of nitroxynil in small intestine (1.1 ± 0.2 μg/mL in wild-type vs. 1.6 ± 0.3 μg/mL in Abcg2^−/−^, *p* = 0.01), a 1.7-fold higher accumulation in spleen (1.4 ± 0.3 μg/mL vs. 2.5 ± 0.3 μg/mL, *p* = 0.001) and an almost 1.6-fold higher concentration in testis (2.6 ± 0.2 μg/mL vs. 4.2 ± 0.8 μg/mL, *p* = 0.02) compared with wild-type mice at 24 h. No significant differences were found in other tissues between both groups of mice. Regarding 96 h sampling time, concentration in most of tissue homogenized samples from wild-type mice, except for the kidney, were below LOQ, but not from Abcg2^−/−^. Brain levels were below LOQ in almost all samples so that they were not included in Table 1. The differential tissue distribution just reported supports the fact that Abcg2 affects systemic exposure to nitroxynil.

### 3.4. Secretion of Nitroxynil into Milk in Wild-Type and Abcg2^−/−^ Mice

**Figure 3:**
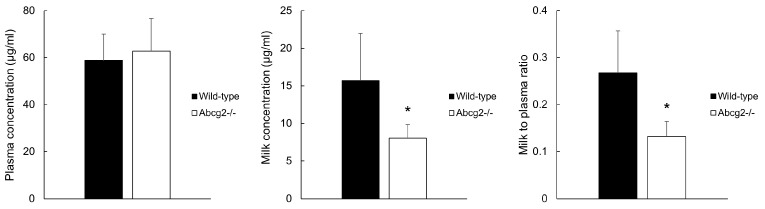
Nitroxynil concentration in plasma and milk samples from wild-type and Abcg2^−/−^ mice 30 min after intravenously administration of 10 mg/kg bw of Distomicide^®^. Milk-to-plasma ratio is included. Results are presented as means and error bars indicate SD. (*) *p* ≤ 0.05, significant differences compared to wild-type mice (Student’s *t*-test, normally distributed data) (*n* = 10–11).

The role of murine Abcg2 in milk secretion of nitroxynil was assessed 30 min after intravenous administration of nitroxynil at 10 mg/kg to lactating wild-type and Abcg2^−/−^ female mice (Figure 3). The nitroxynil plasma concentration was similar in the two experimental conditions (58.8 ± 11.2 μg/mL in wild-type vs. 62.8 ± 13.9 μg/mL in Abcg2^−/−^). Conversely, the milk concentration of nitroxynil was 1.9-fold higher (15.7 ± 6.3 μg/mL vs. 8.1 ± 1. μg/mL, respectively; *p* = 0.004) and milk-to-plasma ratio was 2.1-fold higher (0.3 ± 0.1 vs. 0.1 ± 0.03, respectively; *p* = 0.001) in wild-type mice compared with Abcg2^−/−^ lactating females. Based on our data, we confirm that Abcg2 is involved in the secretion into milk of nitroxynil.

## 4. Discussion

The efficient control of parasitic infections is crucial from both animal welfare and economic perspectives. Thus, anthelmintics are routinely administered to food-producing animals, including dairy animals. Despite the benefits, drug therapy in livestock raises concerns as for public health and food-safety due to the potential risk of unwanted disposition of veterinary drug residues in food products for human consumption, including milk and dairy products. To date, numerous studies have demonstrated the appearance of antiparasitic agents in milk from cattle and sheep, including closantel [25], levamisole [26], ivermectin [27], and nitroxynil [16], among others. For this reason, we consider that the study of factors involved in drug bioavailability and secretion into milk is crucial to optimize the therapeutic use and safety of treatments. In this regard, ABCG2 transporter has been shown to actively participate in absorption, distribution and excretion of different compounds including drugs and endogenous molecules due to its expression in relevant biological barriers [28]. Unfortunately, it also takes part in the secretion of xenobiotics into milk [9]. In this study, we have demonstrated for the first time that the flukicide nitroxynil interacts in vitro and in vivo with the ABCG2 carrier efflux protein. 

We report, through our in vitro results, that nitroxynil is a transported substrate of murine Abcg2 and ovine, bovine and human ABCG2 (Figure 1). Higher transport ratios at 4 h were observed in the murine and ovine subclones compared to human and bovine variants. These differences between subclones have already been reported by González-Sarrías et al. in their in vitro study carried out with urolithin-A and its sulfate conjugate [29]. However, comparison between species variants should be made with caution since although apparent differences between subclones may be due to differences in the affinity/selectivity of ABCG2 species variants for their substrates [30], potential differences in efficiency of ABCG2 transduction cannot be excluded. Thus, our outcomes should only be considered in qualitative terms. 

As nitroxynil is a widely used parasiticide in ruminants, the practical relevance derived from our in vitro outcomes in subclones transduced with the ovine and bovine variants of ABCG2 is mainly based on the management of its therapeutic use and further studies in livestock will be necessary to assess this point. However, the fact that nitroxynil is an in vitro substrate of cells transduced with the human variant is also relevant since human fascioliasis has increased as a public health problem during last few decades, occasionally causing large-scale epidemics with long-term complications such as anemia and malnutrition [31,32]. Although triclabendazole is the most common drug used in the treatment of fascioliasis in humans, reports of resistant infections will make it necessary to approve new treatments, such as nitroxynil. Furthermore, regarding its food safety, residual nitroxynil in edible animal products may lead to severe adverse effects in human health [33] and the activity of ABCG2 in humans could affect this concern. 

In addition, positive results in murine Abcg2-transduced subclones indicate the possible use of mouse as a preclinical model in this case. Previous Abcg2 expression studies in mice revealed high expression in kidney and more moderate in liver, intestine and other organs [34]. Previous plasma pharmacokinetics studies in mice showed that Abcg2 restricts the systemic exposure to xenobiotics, reinforcing the protective role of this transporter against harmful compounds [35]. In this work, we showed lower plasma levels in wild-type compared with Abcg2^−/−^ mice after subcutaneous administration of nitroxynil at all experimental points (Figure 2). Nitroxynil levels were also measured in tissues at 24 and 96 h post-treatment (Table 1). In all tissues, higher accumulation of nitroxynil were reported in the absence of Abcg2 with particular relevance at 96 h, when almost all samples from wild-type mice were below LOQ, but not the ones from Abcg2^−/−^ mice. Although these results may be partially related to the differences in plasma concentrations caused by Abcg2 (Figure 2), the involvement of local Abcg2 in diminished excretion of nitroxynil cannot be discarded. In fact, differences in tissue/plasma ratio were observed in the kidney in the wild-type mice compared to their Abcg2^−/−^ counterparts at 96 h (0.06 ± 0.01 vs. 0.03 ± 0.01, respectively; *p* < 0.001). Besides these data in kidney, our results report a higher accumulation of nitroxynil in the liver and the small intestine of mice not expressing the transporter (Table 1). Considering that the ABCG2 efflux transporter is suggested to be responsible for protecting the body from xenobiotics, we hypothesize that the difference observed in the plasma pharmacokinetics of the Abcg2^−/−^ mice is due to a lower renal, hepatic and/or intestinal clearance mediated by this efflux carrier protein. Bearing this in mind, and considering that, although this drug is metabolized, unchanged drug is likely to be the only source responsible for nitroxynil action, our results are then relevant from the point of view of the flukicide action of the compound. For instance, any mechanism involved in limiting the activity of the transporter would reduce the presence of nitroxynil in the bile ducts, which are known to be the preferential site for adult liver fluke infection [36]. Furthermore, these findings are of great importance since it has been shown that after nitroxynil intoxication, the main lesions occurred in the liver (hemorrhagia and hepatocyte degeneration) and the kidneys (tubular nephrosis) [37]. Our results obtained from testis reveal that Abcg2 also affects testicular accumulation of nitroxynil. This difference had already been described by our research group for other substrates, such as tolfenamic acid showing an almost 3-fold higher accumulation of the anti-inflammatory compound in Abcg2^−/−^ testis compared to wild-type [38]. However, further studies would be required to determine the potentially dangerous effect derived from a greater testicular exposure to the drug.

Despite the treatment with nitroxynil being strongly regulated, the potential risk to dairy consumers cannot be ruled out particularly considering that nitroxynil residues have been found to be extremely persistent in milk compared to other anthelmintic compounds [16]. Unexpected drug residues in food could cause resistance, hypersensitivity, teratogenicity, and changes in the intestinal microbiota of the exposed living organisms, including humans [39]. Remarkably, nitroxynil residues have been found in human breast milk from mothers untreated with nitroxynil [19]. For this reason, the role of Abcg2 in nitroxynil milk secretion was analyzed in this work. Our data support the fact that Abcg2 is an important factor in the secretion of nitroxynil into milk, as indicated by the 1.9-fold higher milk concentration and 2.1-fold higher milk-to-plasma ratio in wild-type compared with the Abcg2^−/−^ female mice 30 min after intravenous administration of the drug (Figure 3). Considering that maternal milk is essential for the optimum growth and development of newborn mammals and that it is an important component of human diet worldwide, ABCG2-mediated transfer of nitroxynil into the milk poses a risk to breast-fed infants and dairy consumers. On the other hand, the presence of this flukicide in a non-targeted manner in the dairy products may increase the risk of anthelmintic resistance development. In this regard, it is worth noting that currently, in the absence of new drugs, parasite control is sometimes exclusively becoming reliant on synergistic combinations of existing compounds to increase the spectrum of activity and efficacy against parasites. In the case of nitroxynil, the combination with ivermectin has been studied for this purpose [16,23]. Inasmuch as ivermectin has been described as an inhibitor of Abcg2 that can affect milk secretion of ABCG2 substrates such as the antimicrobials danofloxacin and meloxicam [21,40], its combination with nitroxynil may affect its secretion into milk. However, further studies to confirm this hypothesis should be carried out. Furthermore, differences in ABCG2 activity due to inhibition by diet compounds such as flavonoids [41,42] or drugs or genetic ABCG2 polymorphism [3,43], not only in the mother but also in the infant, could vary the effective exposure in newborn and adults, considering also age-related changes in ABCG2 abundance [44]. 

Interestingly, no differences in plasma concentration between wild-type and Abcg2^−/−^ female mice were reported (Figure 3), while differences in plasma concentrations were showed in the case of male mice (Figure 2). Although this discrepancy could be attributed to the different route of administration used, sex differences in the effect of Abcg2 in plasma pharmacokinetics of its substrates have been previously described and have been attributed to higher Abcg2 hepatic expression and subsequent higher Abcg2-mediated hepatobiliary excretion in males compared to females [45]. Further studies are needed to confirm this hypothesis in the case of nitroxynil.

To conclude, our results support the fact that ABCG2 is an important factor involved in the pharmacokinetics, tissue distribution and milk secretion of nitroxynil, increasing systemic drug exposure and the appearance of residues in milk. Furthermore, in vitro results demonstrate that this transporter plays a crucial role in the active in vitro transport of nitroxynil in murine, ovine, bovine and human ABCG2-transduced subclones, suggesting that the data obtained in mice may be extrapolated to other relevant species.

## Figures and Tables

**Table 1:** Tissue concentration expressed as µg nitroxynil/g tissue in wild-type and Abcg2^−/−^ male mice at 24 and 96 h after subcutaneous administration of a single dose of Distomicide^®^ at 10 mg/kg (*n* = 4–5).

	24 h			96 h		
	Wild-Type	Abcg2^−/−^	*p* Value	Wild-Type	Abcg2^−/−^	*p* Value
Liver	0.9 ± 0.3	1.3 ± 0.3	n.s.	<LOQ	0.7 ± 0.2 *	–
Kidney	5.9 ± 1.2	8.2 ± 1.8	n.s.	0.9 ± 0.1	3.5 ± 0.3 *	<0.001
Small intestine	1.1 ± 0.2	1.6 ± 0.3 *	0.013	<LOQ	0.5 ± 0.1 *	–
Spleen	1.4 ± 0.3	2.5 ± 0.3 *	0.001	<LOQ	<LOQ	–
Testis	2.6 ± 0.2	4.2 ± 0.8 *	0.021	<LOQ	1.1 ± 0.2 *	<0.001

Results are presented as means ± SD. *: *p* ≤ 0.05, significant differences compared to wild-type mice (Student’s *t*-test, normally distributed data; except for testes (not normally distributed, Mann–Whitney U test)). <LOQ: sample concentration below limit of quantification. n.s.: not significant.

## Data Availability

The data supporting the reported results are available from the corresponding author upon request.
